# Bioactive Hydroxyapatite Aerogels with Piezoelectric Particles

**DOI:** 10.3390/biomimetics9030143

**Published:** 2024-02-27

**Authors:** Catarina Tavares, Tânia Vieira, Jorge C. Silva, João P. M. R. Borges, M. Carmo Lança

**Affiliations:** 1 CENIMAT|i3N, Department of Materials Science, School of Science and Technology, NOVA University Lisbon, 2829-516 Caparica, Portugal; cdc.tavares@campus.fct.unl.pt (C.T.); mcl@fct.unl.pt (M.C.L.); 2 CENIMAT|i3N, Department of Physics, School of Science and Technology, NOVA University Lisbon, 2829-516 Caparica, Portugal; ts.vieira@fct.unl.pt

**Keywords:** piezoelectricity, aerogel, hydroxyapatite, barium titanate, solvothermal synthesis, bioactivity

## Abstract

Open-cell foams based on hydroxyapatite (HAp) can mimic the extracellular matrix (ECM) to better replace damaged hard tissues and assist in their regeneration processes. Aerogels of HAp nanowires (NW) with barium titanate (BT) particles were produced and characterized regarding their physical and chemical properties, bioactivity, and in vitro cytotoxicity. Considering the role of piezoelectricity (mainly due to collagen) and surface charges in bone remodeling, all BT particles, of size 280 nm and 2 and 3 µm, contained BaTiO_3_ in their piezoelectric tetragonal phase. The synthesized nanowires were verified to be AB-type carbonated hydroxyapatite. The aerogels showed high porosity and relatively homogeneous distribution of the BT particles. Barium titanate proved to be non-cytotoxic while all the aerogels produced were cytotoxic for an extract concentration of 1 mg/mL but became non-cytotoxic at concentrations of 0.5 mg/mL and below. It is possible that these results were affected by the higher surface area and quicker dissolution rate of the aerogels. In the bioactivity assays, SEM/EDS, it was not easy to differentiate between the apatite deposition and the surface of the HAp wires. However, a quantitative EDS analysis shows a possible CaP deposition/dissolution cycle taking place.

## 1. Introduction

Bone is a dynamic tissue capable of regenerating, growing, and remodeling to preserve its structural integrity and mineral homeostasis. While the natural remodeling ability of bone tissue is remarkable, there exists a threshold limit that dictates the speed and extent to which regeneration can occur. For instance, fractures due to traumatic injuries, age degenerative processes, or even surgical removal of tumors that result in the absence or large defects of bone, lead to the need for new features of biomaterials and medical devices to improve the natural healing process of bone [1,2]. The increasing importance of orthopedic biomaterials is reflected in the growth of their global market. In 2022 this market reached around USD 17,000 million and it is expected to grow by about 10% in 2023 [3]. From these numbers, it is evident that there is a constant demand for innovative biomaterials in this field so that patients’ healing ability is improved and the time of recovery can be reduced. Materials that enhance osteointegration and osteogenesis, particularly in the early stages of bone regeneration, will help to reduce the period of patients’ immobilization.

One material of interest is hydroxyapatite (HAp), represented by the chemical formula Ca_10_(PO_4_)_6_(OH)_2_. This ceramic belongs to the family of calcium phosphates (CaP), also named apatites. It is a highly biocompatible ceramic, chemically similar to the bone’s mineral phase, and natural HAp that has a Ca/P atomic ratio between 1.5 and 1.67 and demonstrates osteoconductive, osteoinductive, and osteointegrative properties [4,5]. The main mechanism for bioactivity is the release of ionic products, such as calcium and phosphate ions, by partial dissolution upon implantation. This process, in turn, leads to the precipitation of biological apatite on the surface of the ceramics in a process called bone mineralization. Consequently, this contributes to the attachment of the implant to the host’s bone, improving implant fixation to the surrounding tissues [4,6]. Since HAp bioceramics are brittle in nature and have low fracture toughness compared to natural bone, their applications in orthopedics are usually as non-load-bearing implants, the filling of bone defects, or in load-bearing implants as coatings, either in dense or porous forms [6].

In addition to the chemical composition of the scaffolds, their morphology also plays an important role in the process of bone remodeling and the osteointegration of the implant [7]. For instance, the porosity of a scaffold, controlled by its synthesis method, has been shown to improve implant performance due to better bio-integration and mechanical stability of the implant. The higher surface area and open pore structure improve the transport of oxygen and nutrients, along with higher surface area enhancing the migration of cells responsible for bone tissue deposition [5,8].

Among various HAp morphologies, hydroxyapatite nanowires (HAp NWs) seem to be highly promising in several fields [9,10,11]. Huang et al. explored the use of highly porous aerogels (~99.7% porosity) composed of HAp NWs as scaffolds in bone regeneration and neovascularization [11]. Compared to dense hydroxyapatite, the HAp NWs aerogels promote adhesion and migration deeply into the pores of osteoblasts and other cells responsible for bone regeneration. Moreover, these aerogels exhibit better mechanical properties, such as high elasticity and high fracture resistance due to the flexibility and 3D network of the HAp nanowires [9,10].

Barium titanate (BaTiO_3_, BT) is a perovskite ceramic with interesting applications in the biomedical field. In its tetragonal phase, it demonstrates piezoelectric properties and is also biocompatible [12]. As early as 1957, Eiichi Fukada et al., developed research that proved the existence of piezoelectricity in bone [13,14]. Currently, it is now known that piezoelectric effects dominate in dry bone, while in wet bone, regeneration and remodeling electric cues are mainly related to stress generated potentials (SGPs), with a smaller contribution of the direct piezoelectric effect [15,16]. Therefore, surfaces that present stable electrical charges, either by dipole orientation or space charge trapping, can accelerate the regenerative processes of hard tissue [17]. Implementing this surface modification in biomaterials, using materials with permanent dipoles, such as tetragonal BT, and mimicking the natural behavior of bone tissue seems quite helpful as a stimulus for bone remodeling, possibly enhancing the osteoinduction effects of the implant. As highlighted in the recent literature review by Zhang et al. [18], there are an increasing number of studies to enhance the bone regenerative response using composites of HAp and BT, including developments presenting open porous morphology. Also, recently, interest has increased in highly structured HAp materials to overcome the brittleness typical of ceramics [19]. In these are included the aerogels of nanowires of HAp as prospective biomaterials in hard tissue engineering [20,21]. To the best of the authors’ knowledge, aerogels of HAp nanowires/BT micro- and nanoparticles are a novelty that attempts to combine the advantages of a highly porous structure as a cell scaffold (better osteoconductitvity) and to accelerate osteogenesis and osteoinduction by the presence of surface charges induced by the piezoelectric BT.

The main purpose of this work was the synthesis and physical, chemical, and in vitro biological characterization of a hydroxyapatite aerogel along with an innovative composite aerogel of HAp embedded with barium titanate particles with significant potential for biomedical applications. All the BT particles and the aerogels were characterized by X-ray diffraction (XRD), Fourier-transform infrared (FTIR), and Raman spectroscopies. The HAp aerogels were analyzed by SEM to make sure that the pore size and interconnectivity of pores were suitable for their expected applications. In addition, the dispersion of the BT particles was observed on the composite aerogels. The biocompatibility of all materials was evaluated by cytotoxicity assays. Finally, the bioactivity of the aerogels with and without the piezoelectric particles was studied through simulated body fluid (SBF) immersion.

## 2. Materials and Methods

### 2.1. Barium Titanate

Commercial tetragonal BT particles with sizes of 280 nm (LT#NG04MO0503—Nano-grafi, Jena, Germany), 2 µm (LT#MKBB0111V—Sigma-Aldrich, Saint Louis, MO, USA), and 3 µm (LT#MFCD00003447—Sigma-Aldrich) were selected because of their piezoelectricity. Additionally, the selection aimed to test the effect of different particle sizes on bioactivity.

### 2.2. Hydroxypatite-Based Aerogels

The HAp NWs were synthesized based on previous research by Zhi-Chao Xiong et al. [9]. Firstly, a solution of 9.36 g of oleic acid (31997—Alfa Aesar, Kandel, Germany) in 13.50 g of deionized water and 4.75 g of methanol (L13255—Alfa Aesar) was mixed under mechanical stirring. A second solution consisting of 1.05 g of NaOH (reagent grade, 97%—655104—Sigma-Aldrich) dissolved in 15 g of deionized water was poured into the first solution and stirred for 30 min, while two other solutions of 0.33 g of CaCl_2_ (anhydrous, 93%—12316—Alfa Aesar) dissolved in 12 g of deionized water and 0.94 g of NaH_2_PO_4_·2H_2_O (purum p.a., ≥99.0%—71500—Sigma-Aldrich) dissolved in 18 g of deionized water were separately poured into the previous mixture and stirred for 10 min each. Finally, 5 mL of the reaction system was poured into a 100 mL Teflon-lined stainless-steel autoclave for a solvothermal reaction. This reaction was tested for different times such as 5, 7, 18, and 24 h at temperatures of 120, 165, and 180 °C. The resulting solution was stirred for 30 min at 300 rpm. To separate the HAp NWs from the impurities, the solution was centrifuged for 10 min at 3000 rpm and washed with progressively diluted methanol/deionized water ratios. The HAp NWs were stored in deionized water.

Following this, the best solvothermal conditions for the formation of HAp NWs were chosen by observing the previously synthesized and dried HAp NWs under SEM and by XRD characterization. The synthesis parameters selected, 180 °C and 18 h, yielded more differentiated and longer nanowires (see Section 3.2).

Next, four different slurries were made from the solution of HAp NWs synthesized at 180 °C for 18 h and stored in deionized water, where one contained only the HAp NWs and three corresponded to 20% BT/80% HAp (ratio of *w*%/*w*%), using tetragonal BT particles with sizes, respectively, of 280 nm and 2 and 3 µm, dispersed through sonication for 15 min. To produce the aerogel structures, each slurry was put into a mold and frozen at –5 °C for 12 h, followed by lyophilization at −40 °C and 0.001 mbar for 24 h.

The HAp aerogel without BT particles was denominated HAp, and the three HAp aerogels with BT were named, respectively, HAp/BT280 for an aerogel embedded with 280 nm sized particles, HAp/BT2 in the case of 2 µm, and HAp/BT3 for the aerogel with the 3 µm particles.

### 2.3. Materials Characterization

#### 2.3.1. Structural, Chemical, and Morphologic Analysis

The analysis of the crystallographic structures was conducted in an X’Pert PRO (PANAnalytical, Malvern, UK) X-ray diffractometer with the use of CuKα radiation generated at 45 kV and 40 mA, in the range of 15° < 2θ < 60°, with a step size of 0.08° for the Hap NWs and in the range of 20° < 2θ < 80°, with a step size of 0.08° for the BaTiO_3_, particles. An additional analysis of the BT, in order to better observe the phase composition of the sample, was conducted in the range of 44° < 2θ < 47°, with a step size of 0.002°.

Additionally, for BT and HAp, a FTIR analysis with the use of the spectrophotometer FT-IR Thermo Nicolet 6700, was carried out in the wavenumber range from 4000 to 400 cm^−1^ to identify the functional groups of BT and HAp. To evaluate the molecular structure of the samples and to confirm the presence of the tetragonal phase in BaTiO_3_, a Raman spectroscopy analysis was conducted using the Renishaw (Wotton-under-Edge, UK) inVia Qontor micro-Raman spectrometer. Five scans were made, each with an integration time of 1 s, using an incident 632.81 nm laser with an intensity of 0.32 mW and a frequency range from 150 to 1000 cm^−1^.

To ensure the existence of HAp nanowires, the aerogel’s morphology, and the dispersion of the BT particles within them, the samples were observed under a scanning electron microscope/Energy Dispersive X-ray Spectrometry (SEM/EDS), model Hitachi (Tokyo, Japan) TM 3030 Plus. All samples were sputter-coated with either gold or titanium before SEM observation.

#### 2.3.2. Biocompatibility Assays

The colorimetric cytotoxicity assays, with the use of resazurin, were performed on all the BaTiO_3_ particles and HAp aerogels, with and without BT, in conformity with the standard ISO-10993-5 “Biological evaluation of medical devices, Part 5: Tests for in vitro cytotoxicity” test protocol [22]. All samples subjected to these tests were previously sterilized by heating at 150 °C for 5 h. Extracts of the BT particles were prepared by leaving them in contact with a complete culture medium (McCoy 5A from Sigma Aldrich supplemented with 10% fetal bovine serum from Biowest and 1% penicillin/streptomycin from Gibco, Waltham, MA, USA) at a concentration of 60 mg/mL at 37 °C for 48 h, while the extracts of aerogels were prepared in complete culture medium at a concentration of 1 mg/mL at 37 °C for 48 h. Additionally, SaOs2 cells (provided by the American Type Culture Collection, ATCC, Manassas, VA, USA HTB-85) were trypsinized using TrypLE Express (from Gibco) for 5 min at 37 °C and the cells were counted using a hemocytometer. The cells were seeded at a density of 30,000 cells/cm^2^ in 96-well plates and incubated at 37 °C and 5% CO_2_ for 24 h. After that period, the culture medium was replaced by the previously mentioned extracts (initial concentration C0) and 3 dilutions (C0/2, C0/4, and C0/8). A positive control (cytotoxic), where cells were treated with 10% dimethyl sulfoxide (DMS), and a negative control (non-cytotoxic), where cells were treated with a complete culture medium, were defined. The cells were left in contact with the extracts for 48 h. Following that, the medium was replaced by a solution containing 50% resazurin (B21187—Alfa Aesar) solution at 0.04 mg/mL in PBS and 50% complete culture medium, and the plates were incubated for 3 h at 37 °C and 5% CO_2_. After that time, using a Biotek (Winooski, VT, USA) ELX800 microplate reader, the absorbance was read at 570 and 600 nm and the corrected absorbance was determined by subtracting the absorbance measured at 600 nm from the one measured at 570 nm and subtracting the medium control. Cell viability is given by the percentage of viable cells in the tested samples relative to the negative control.

#### 2.3.3. Bioactivity Assessment

The bioactivity of the HAp and HAp/BT aerogels was analyzed by evaluating the growth of CaP structures on the surface of the membrane when in contact with simulated body fluid (protocol for SBF solution can be found in Supplementary Materials). The samples evaluated consist of a HAp aerogel without particles, three HAp aerogels with BaTiO_3_, each with a different particle size, and a control group that was not in contact with SBF. Each sample was submerged for 1, 3, and 7 days and the SBF solution was renewed every 2 days. After removal from SBF, the samples were rinsed in distilled water to remove water-soluble salts, such as NaCl. Finally, the samples were observed in SEM and EDS to identify the growth of CaP structures.

## 3. Results and Discussion

### 3.1. Barium Titanate

#### 3.1.1. X-ray Diffraction Analysis

**Figure 1:**
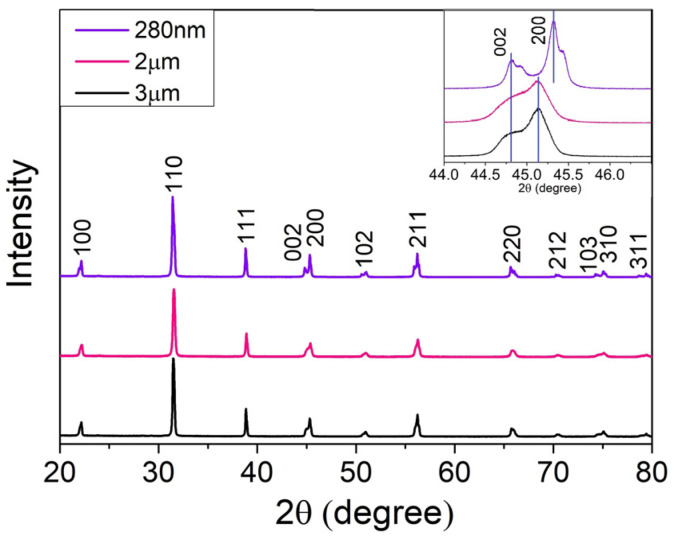
XRD analysis of the BaTiO_3_ powders.

The XRD analysis was carried out to identify the tetragonal phase of the BaTiO_3_ particles and its prevalence over the non-piezoelectric cubic phase. Comparison of the peaks with the ICDD data sheets #00-005-0626 and #01-084-9618, corresponding to tetragonal and cubic BT, respectively, confirmed that the diffractograms in Figure 1 are characteristic of BT in the tetragonal phase (also, no additional peaks due to impurities were detected).

The transformation from cubic to tetragonal phase exhibits itself in the XRD as the presence of double peaks along the diffraction pattern due to the asymmetry of the unit cell along the *c* axis [23]. For BaTiO_3_, this is seen in better detail around 2θ = 45°, corresponding to planes (002) and (200), where the existence of a split peak distinguishes tetragonal BT from its cubic phase. The double diffraction peak is more visible on the 280 nm powders, with a peak at 44.8° (002) and 45.4° (200). Regarding the 2 and 3 µm powders, there is a similarity between the intensity of the diffraction split, also corresponding to the same planes. As can be observed, when compared with the nanometric BT, the peaks’ separation is much less pronounced for the micrometric particles. These results are in agreement with the DSC data for BT 280 nm powders (the DSC/TG plot is presented in Supplementary Materials in Figure S1), where a small peak is observed at 130.6 °C, the Curie temperature, corresponding to the transition from tetragonal to cubic BT. On the contrary, no peak could be perceived for the 2 and 3 µm particles in the DSC data.

The average sizes of the crystalline grains, *τ*, present in Table 1, were calculated according to the Scherrer Equation (1), using an X-ray wavelength of *λ*(*CuKα*) = 1.5418 Å, a shape factor of the crystallite, *κ*, of 0.9, and a line broadening at half the maximum intensity, *β*, for each peak [24,25,26]:

(1) $$\tau =\frac{\kappa \lambda }{\beta \cos\theta }$$

As can be seen, the average crystallite sizes are larger for the micrometric samples, 2 and 3 µm, leading to larger, less defined peaks and higher superposition between each other. Although all the samples seem to exhibit a tetragonal-dominant structure, as stated before, the samples of 2 and 3 µm show a smaller difference between the angles corresponding to the two peaks in Figure 1, when compared to the sample of 280 nm, which is caused by a reduced tetragonal phase and the partial existence of a cubic phase. A higher percentage of tetragonal phase, such as in the particles of 280 nm, would be more suited for a possible polarization of the HAp aerogels [18].

#### 3.1.2. Fourier-Transform Infrared Spectroscopy Analysis

**Figure 2:**
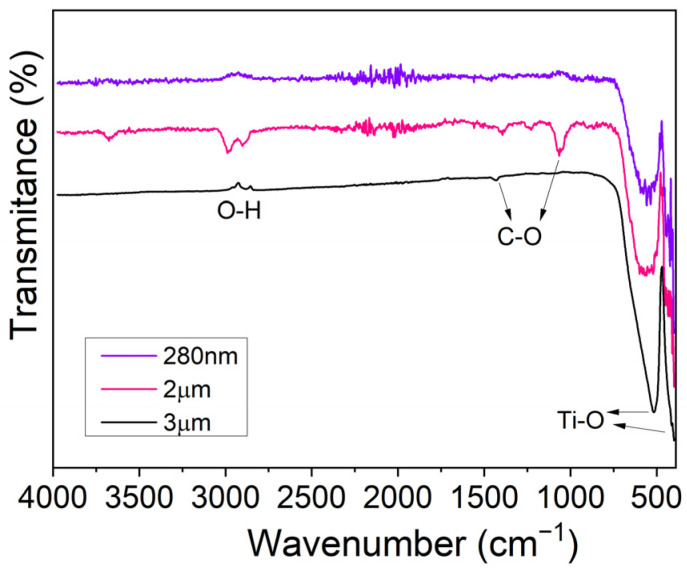
FTIR spectra of 280 nm and 2 and 3 µm BaTiO_3_ particles, measured in transmittance mode.

Complementary to the XRD structural analysis, FTIR spectra of the commercial BaTiO_3_ particles were obtained, allowing us to identify the presence of functional groups of BT and to identify bonds that are related to synthesis by-products or residual reactants. In this way, possible compounds that might affect the purity and relevant properties of BT were identified. The FTIR spectra in Figure 2, measured in transmittance mode, exhibit sharp peaks at 438 cm^−1^ and around 540 cm^−1^, indicating the existence of a Ti-O bond, characteristic of BT [27,28]. Moreover, vibrational bands signaled at 860 and 1450 cm^−1^ are compatible with bending vibrations of a C-O bond, which could be linked to the reagents used for the fabrication of the commercial particles, for instance, barium carbonate.

Finally, for nanometric BT, the broad band around 3000 cm^−1^ can be attributed to the presence of an OH group from the H_2_O content in the samples according to Singh et al. [28]. In the same region, we can see two peaks for the micrometric BT particles from around 2900 to 3000 cm^−1^, which are associated with Ba-OH due to a possible incorporation of OH^−^ in the lattice [27,28], which can affect the tetragonal lattice and, therefore, piezoelectricity [29].

#### 3.1.3. Raman Spectroscopy Analysis

**Figure 3:**
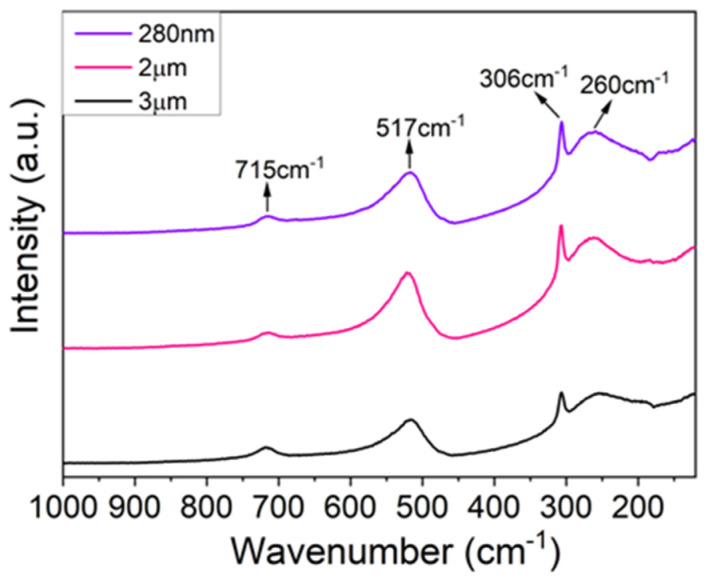
Raman spectroscopy of BaTiO_3_ particles with sizes 280 nm and 2 and 3 µm.

To further confirm the existence of a tetragonal phase of the powders, an analysis of the powder’s Raman spectroscopy was used. A molecule of BT contains five atoms, leading to 12 optical vibrational modes. Based on the crystallography, Raman-active modes for tetragonal BT are four E(TO + LO), three A_1_(TO + LO), and a B_1_(TO + LO), while no Raman-active mode is predicted for the cubic phase due to the isotropic distribution of electrostatic forces surrounding Ti^4+^ ions [30]. Accordingly, the Raman spectra presented in Figure 3 are typical of a nonsymmetric structure.

In the Raman spectra, four dominant bands centered near 260, 306, 517, and 715 cm^−1^, indicating the presence of the tetragonal phase, can be identified. As the literature suggests, the broad Raman band near 260 cm^−1^ corresponds to a Raman mode A_1_(TO_2_), and a band near 306 cm^−1^ is assigned to the B_1_ and E(TO + LO) modes, suggesting an asymmetric vibration of the [TiO_6_] octahedra. In addition, the band at 517 cm^−1^ is related to the A_1_(TO_3_) and E(TO) modes, while a Raman band around 715 cm^−1^ is correlated with the A_1_(LO) and E(LO) modes [23,30]. Accordingly, the Raman results clearly show that all BaTiO_3_ samples possess a distortion of the [TiO_6_] octahedra and, consequentially, ferroelectric properties [23] in agreement with XRD and FTIR data presented before.

### 3.2. Hydroxyapatite Nanowires

#### 3.2.1. SEM Analysis

During the synthesis of HAp NWs, the chemical reactions that take place between the reactants are as follows [31]:

C_18_H_34_O_2_ + NaOH → C_18_H_33_O_2_Na + H_2_O (2)

2C_18_H_33_O_2_Na + CaCl_2_ → (C_18_H_33_O_2_)_2_Ca + 2NaCl (3)

NaH_2_(PO_4_)·2(H_2_O) + 2OH^−^ → PO_4_^3−^ + 4H_2_O + Na^+^ (4)

10(C_18_H_33_O_2_)_2_Ca + 6PO_4_^3−^ + 2OH^−^ → Ca_10_(PO_4_)_6_(OH)_2_ + 20C_18_H_33_O_2_ (5)

It can be noticed that the calcium oleate acts as both the calcium source and the precursor for the formation of HAp NWs, while NaH_2_PO_4_ acts as the phosphorus source. At the beginning of the reaction system, calcium oleate is formed in chemical reactions (2) and (3). In chemical reaction (4), sodium dihydrogen phosphate dihydrate is hydrolyzed to form PO_4_^3−^ ions. During the crystallization process in reaction (5), hydroxyapatite nuclei are formed under solvothermal conditions. These nuclei later grow into longer structures, in a relatively long period of time, under relatively high temperatures and pressures, as previously mentioned in Section 2.

The existence of nanowires was first confirmed by observing, with SEM, the resulting HAp slurry, after being dried at 40 °C. For synthesis temperatures of 120 °C (data presented in Supplementary Figure S2), no structures were present for either 24 or 30 h of synthesis. Probably, the temperature is too low for the occurrence of the solvothermal reaction. Therefore, this solvothermal synthesis temperature was automatically rejected for the formation of HAp NWs.

**Figure 4:**
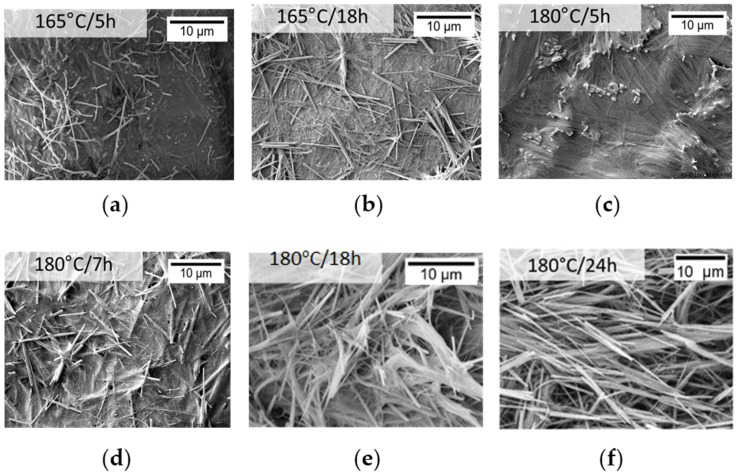
SEM imaging of hydroxyapatite synthesized at (**a**) 165 °C for 5 h; (**b**) 165 °C for 18 h; (**c**) 180 °C for 5 h; (**d**) 180 °C for 7 h; (**e**) 180 °C for 18 h; (**f**) 180 °C for 24 h.

Higher temperatures, such as 165 and 180 °C, were evaluated with synthesis times of 5, 7, 18, and 24 h, with resulting samples presented in Figure 4. For shorter synthesis times, such as 5 h, exhibited in Figure 4a,c for 165 and 180 °C, respectively, several structures appear to be starting to form; however, they are almost completely blended with each other. Only in Figure 4d, at 7 h, do some longer structures appear in the vicinity of more differentiated rodlike structures, with lengths around 6 µm. However, these longer structures also appear merged, preventing differentiation from each other. In the case of longer synthesis times, the sample of Figure 4f, of 24 h and 180 °C, shows mostly rod structures with around 10 µm length, whereas for 18 h, both samples, in Figure 4b,e, produced at 165 °C and 180 °C, respectively, display rodlike structures surrounded by wires. However, it should be noted that, with temperatures of 165 °C, the wires appear to be somewhat merged similarly to the sample of 7 h and 180 °C, present in Figure 4d. 

#### 3.2.2. X-ray Diffraction Analysis

Following the evaluation of the NWs morphology, some of the solvothermal synthesis temperatures were discarded. Therefore, an evaluation of the crystallinity of HAp and the existence of other compounds was conducted on the samples represented in Figure 4: 165 °C (5 and 18 h) and 180 °C (5, 7, 18, and 24 h). To evaluate the existence of HAp and other impurity compounds, the diffraction peaks were compared to the ICDD sheet of hexagonal Ca_10_(PO_4_)_6_(OH)_2_ and JCPDS cards of reagents used during the synthesis, such as CaCl_2_, NaH_2_PO_4_·2H_2_O, and NaOH.

**Figure 5:**
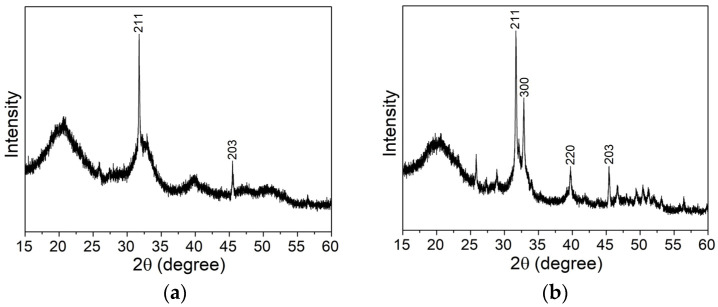
XRD diffractogram for HAp NWs synthesized for 5 h at (**a**) 165 °C and (**b**) 180 °C.

Firstly, for either temperature, a synthesis time of 5 h does not lead to the formation of crystalline hydroxyapatite as there is still the presence of wide peaks and humps, more noticeable in Figure 5a, typical of an amorphous structure. The position of the XRD hump in amorphous HAp is around 30°, while in the obtained pattern, a hump is clearly visible at around 20° and it is also possible to perceive a small hump around 30°. The 20° hump could be related to amorphous dicalcium phosphate anhydrous, DCPA (monetite), and CaHPO_4_, according to Borkiewicz [32]. However, both XRD diffractograms in Figure 5 exhibit some characteristic crystalline HAp peaks, such as those around 31.7° and 45.4°, that can indicate the beginning of HAp synthesis. It can be noted that, for the same synthesis time, more HAp characteristic peaks were formed at a higher temperature of 180 °C, present in Figure 5b, than at 165 °C, in Figure 5a, essentially showing that higher temperatures accelerate the formation of HAp crystallites, as expected.

**Figure 6:**
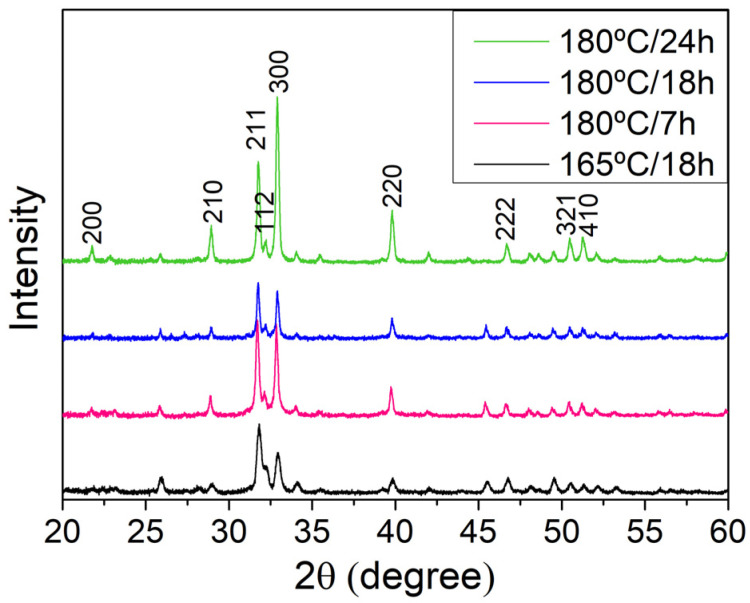
XRD diffractogram for HAp NWs synthesized at different temperatures and times.

The remaining diffraction results, presented in Figure 6, clearly show the presence of crystalline hydroxyapatite in all the samples, with all peaks corresponding to the ICDD datasheet, although some differences between the relative intensities of the identified peaks can be found. For instance, in the case of the sample of 180 °C for 24 h, the two highest intensities, (211) and (300), are switched when compared to tabulated data or other samples in Figure 6. This phenomenon could be explained by a preferential formation of crystalline grains along the longitudinal direction of the nanowires, which appear to be the direction perpendicular to the (300) plane, increasing the intensity of the measured peak at 32.8° and highlighting that change in peak intensities for the larger structures formed for the synthesis time of 24 h. In addition, no characteristic peaks of the reagents or by-products were found in the diffractograms shown in Figure 6.

The chosen synthesis time and temperature for the HAp NWs, which will be used to obtain the HAp aerogels, were 180 °C for 18 h due to both the sample’s XRD diffractograms, typical of crystalline HAp, and the morphology, observed in Section 3.2.1, which seems to present a higher quantity of and longer wirelike structures when compared to the other samples. This more intertwined structure between the HAp wires could be an advantage in the mechanical stability of the aerogels.

The average size of the crystalline grains, *τ*, present in the HAp 180 °C for 18 h sample was calculated to be 39.05 nm, according to Equation (1), where 2*θ* = 31.74°, with *β* of 3.694 × 10^−3^ rad, *λ*(*CuKα*) = 1.5418 Å, and *κ* of 0.9 [24,26,32].

#### 3.2.3. Fourier-Transform Infrared Spectroscopy Analysis

**Figure 7:**
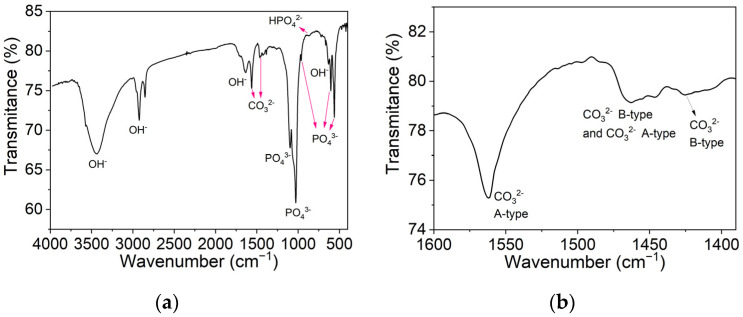
FTIR spectra of HAp synthesized at 180 °C for 18 h for wavenumbers between (**a**) 4000 and 400 cm^−1^; (**b**) 1600 and 1350 cm^−1^.

In Figure 7a, an FTIR analysis of hydroxyapatite synthesized at 180 °C for 18 h is shown. The spectrum shows the typical HAp features, containing sharp O–H and P–O bands. The bands of significant intensity, around 1026 and 1100 cm^−1^, and the bands near 560, 600, and 960 cm^−1^ correspond to a symmetric stretching vibration of the PO_4_^3−^ tetrahedron. Moreover, the band around 633 cm^−1^ is attributed to the stretching modes of hydroxyl groups in HAp [20,32,33].

The small band at 870 cm^−1^ indicates the presence of HPO_4_^2−^ ions. This observation suggests the existence of non-stoichiometric HAp and the presence of carbonated apatite, which can be confirmed by the characteristic bands between wavenumbers 1460 and 1530 cm^−1^. As seen in Figure 7b, two types of substitutions occurred, A-type carbonate substitutions, where carbonate ions substitute hydroxyl groups, and B-type carbonate substitutions, resulting from carbonate ions that have substituted phosphate ions [31,32,33,34]. It can be concluded that the resulting product from the solvothermal synthesis at 180 °C for 18 h is AB-type carbonated apatite. The B-type carbonate substitutions in the apatite lattice are known to increase the extent of solubility in weak acids, a characteristic that can help the substitution of the aerogel with new bone when using this material in bone regeneration [31,32,34]. Adsorbed water can also be seen on the wide bands that appear on the spectra from 2600 to around 3600 cm^−1^ and at 1640 cm^−1^ [33].

### 3.3. HAp and HAp/BT Aerogels

#### 3.3.1. Porosity of the Aerogels

The porosity of the aerogels was calculated using Equation (6), where EW is the expected weight of a HAp solid material, calculated using hydroxyapatite’s theoretical density, 3.16 g/cm^3^, and the average aerogel’s volume of 0.53 cm^3^, and RW represents the aerogel’s real weight, measured for each sample and which is, on average, 5.35 mg [11].

(6) $$P(\%)=\frac{\mathrm{RW}}{\mathrm{EW}}\times 100$$

The samples produced presented an average porosity of (99.68 ± 0.02)% for both HAp and HAp/BT aerogels, with no significant variation between the different aerogel types [11,35].

#### 3.3.2. SEM Analysis

**Figure 8:**
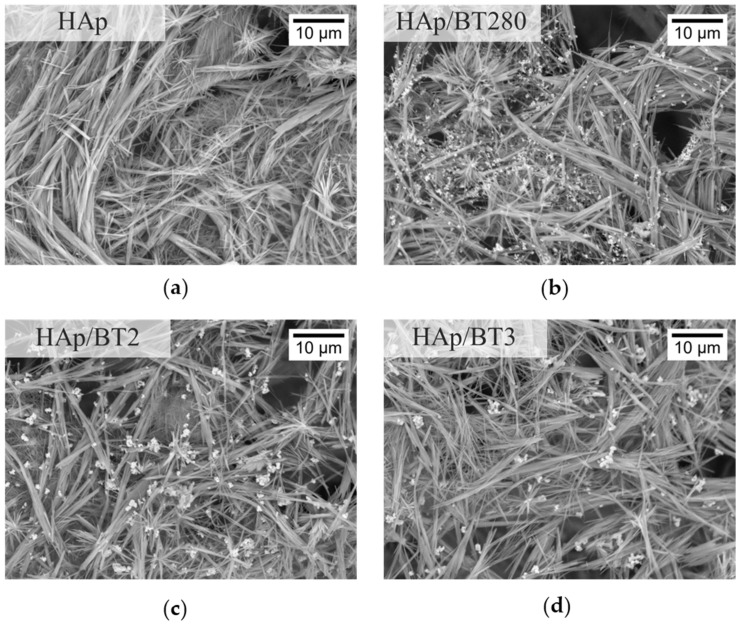
SEM imaging of aerogel samples: (**a**) HAp; (**b**) HAp/BT280; (**c**) HAp/BT2; (**d**) HAp/BT3.

The microscopic homogeneity and porosity of the HAp and HAp/BT aerogels was investigated by imaging the surface of the aerogel using SEM. As can be seen in Figure 8, all samples show similar heterogenous porosity throughout, with pores at a micrometer level, relevant for cell penetration in this scaffold. Furthermore, as can be seen, the aerogels’ 3D network created by the nanowires gives rise to a high surface area. This is further verified by the very high value obtained for the porosity value and, consequently, results in a low density for the aerogel (0.1 mg/cm^3^).

The length of the wires was not possible to measure since, many times, the interwinding prevented the start and/or end of the wire from being visible in the images. Still, some shorter wires, with a length of approximately 10 µm, were observed. The diameter of the wires varied, though it appeared to be less than 1 µm.

Regarding the aerogels with BT shown in Figure 8, the particles appeared to be dispersed evenly throughout the aerogel, although in certain areas there seemed to be some agglomerates.

#### 3.3.3. Cytotoxicity Assays

In vitro colorimetric assays using resazurin, in conformity with the standard ISO-10993-5 test protocol [22], allowed a study of the potential cytotoxic effect of BT particles, of sizes 2 µm, 3 µm, and 280 nm, and of the HAp, HAp/BT280, HAp/BT2, and HAp/BT3 aerogels. Given the low mass of the aerogels, the concentrations chosen were significantly lower, 1 mg/mL, than the ones used for the BT particles, 60 mg/mL.

**Figure 9:**
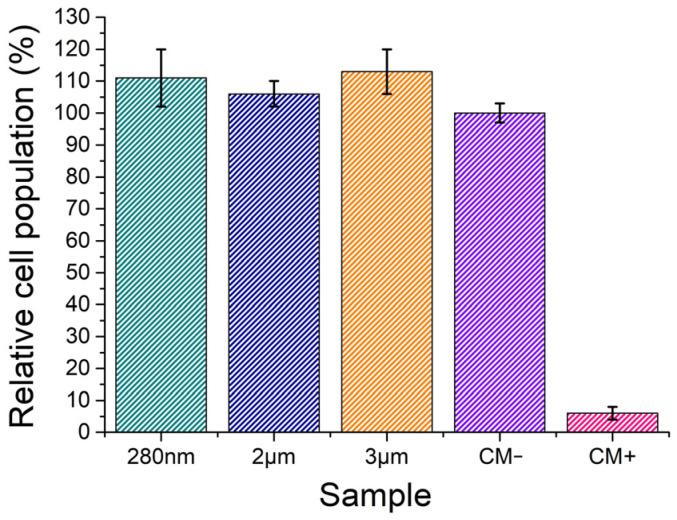
Relative cell populations obtained in the cytotoxicity tests of BaTiO_3_ particles, 280 nm, 2 µm, 3 µm, negative (CM−), and positive (CM+) control groups. Populations obtained using the extracts prepared at a concentration of 60 mg/mL. Results are average and experimental standard deviation.

As seen in the graph in Figure 9, all BT powders revealed high cell viability, with a relative cell population above 100%, which is the result of not only cell survival but also cell proliferation. Therefore, it is safe to assume that the BT powders are non-cytotoxic for all the concentrations studied.

**Figure 10:**
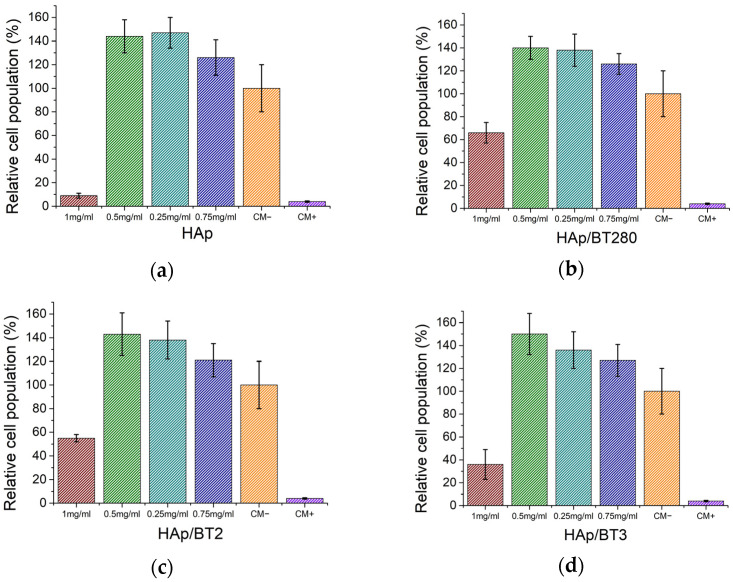
Relative cell populations obtained in the cytotoxicity tests of the aerogels: (**a**) HAp; (**b**) HAp/BT280; (**c**) HAp/BT2; (**d**) HAp/BT3, and negative (CM−) and positive (CM+) control groups. Populations obtained using the extracts prepared at a concentration of 1 mg/mL and three serial dilutions. Results are average and experimental standard deviation.

In the case of the aerogels, the relative cell population for different concentrations of the extracts is shown in Figure 10. The same pattern was attested in every sample. For the highest concentration used (C0 = 1 mg/mL), the HAp and HAp/BT3 samples were considered severely cytotoxic while the other samples were moderately cytotoxic. For subsequent dilutions, C0/2 to C0/8 (concentration range of 0.5–0.125 mg/mL), all samples showed a non-cytotoxic behavior and can be considered biocompatible. On the other hand, the results for the dilutions, showing a relative cell population of 140%, are unusually high to be justified purely by cell viability and proliferation. It could be speculated that the reduction of resazurin is being affected by other parameters and that the cell populations might have been overestimated.

According to Klimek et al. [36], the apparent cytotoxicity of the HAp might be caused by a massive uptake of Ca^2+^ and HPO_4_^2−^ ions from the medium, related to the high surface area of the ceramics, resulting in extracts that are not optimal for cell cultures. This phenomenon will lead to an unexpected cytotoxic effect despite these ion reactions being correlated with increased bioactivity. Moreover, in vivo, tissue liquids continuously circulate around implanted biomaterial and supplement all the adsorbed ions in the implantation area. This could be the case for the ceramics produced, since Gustavsson et al. [37] also reported ion adsorption by calcium-deficient hydroxyapatite with a carbonated surface chemistry, similar to the AB–type carbonated apatite’s chemistry observed in FTIR in the Section 3.2.3. When the extracts’ concentration range is 0.5–0.125 mg/mL, the uptake of ions might not be enough to lead to cell death. Nevertheless, it can still influence the natural behavior of the viable cells, leading them to overproduce enzymes that reduce resazurin and thus obtain an overestimation of the cell population [38].

#### 3.3.4. Bioactivity Assays in Simulated Body Fluid

The bioactivity was analyzed by submerging the aerogels HAp and HAp/BT in an SBF solution and placing them in an orbital shaker at 37 °C, mimicking average body temperature, for 1, 3, and 7 days, and by preparing a control group which was not submerged. All samples were analyzed through SEM/EDS.

**Figure 11:**
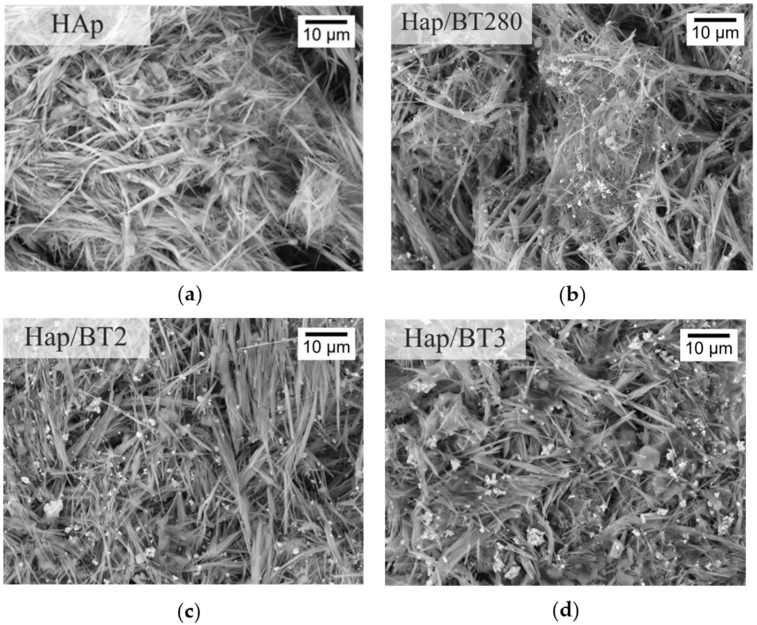
SEM images after 7 days in SBF of the aerogel samples: (**a**) HAp; (**b**) HAp/BT280; (**c**) HAp/BT2; (**d**) HAp/BT3.

In SEM images, there is no clear evidence of the deposition of apatite crystals for all aerogel samples and for all of the immersion times evaluated, as seen in Figure 11, which corresponds to 7 days in SBF. As expected, the EDS mapping data (presented in Supplementary Figures S3 and S4) reveal the existence of Ca and P in the wire structures, barium, and titanium on the BT particles (visible in the agglomerate of Figure S3). There is also some residue of Na and Cl, with some crystals typical of the cubic lattice of sodium chloride present. This residue might hinder the observation of other structures in some areas of the SEM images. As stated in Section 2, all samples were rinsed in distilled water after removal from SBF with the purpose of removing this type of water-soluble residue. However, there are some materials and morphologies that do not allow the full dissolution of NaCl. The rough morphology and chemistry of the sample, which naturally contains CaP, could also be preventing the observation of deposited apatites in the case of relatively small crystals being formed.

Regarding the calcium phosphate deposition on the BT particles, from the SEM imaging in Figure 11, it can also be noted that, after 7 days, no apparent apatite deposition occurred around the barium titanate particles present in the aerogel (EDS mapping presented in Supplementary Materials Figure S3).

Since the SEM imaging and EDS mapping do not confirm if CaP deposition occurred, an EDS analysis was used, and the Ca/P ratio for all samples was estimated to observe if a trend in Ca^2+^ and PO_4_^3−^ deposition was occurring.

**Figure 12:**
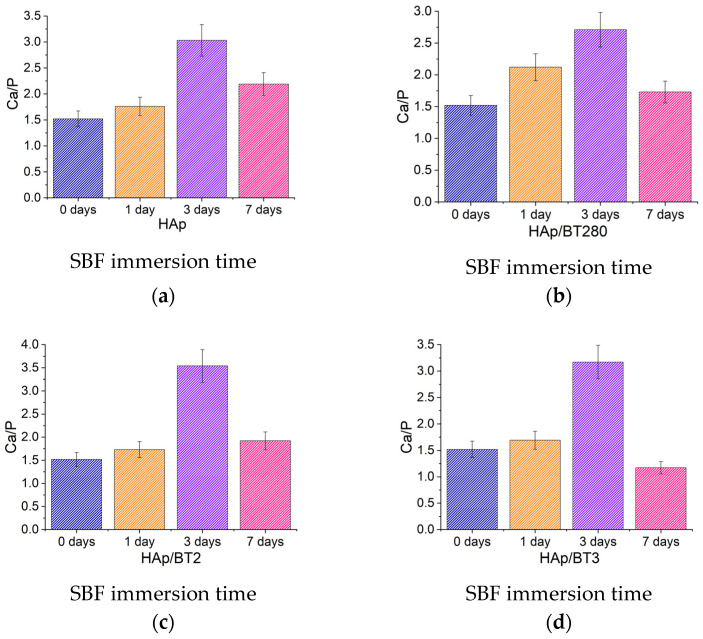
Ca/P, calculated by quantifying the EDS analysis after SBF immersion of the (**a**) HAp; (**b**) HAp/BT280; (**c**) HAp/BT2; and (**d**) HAp/BT3.

In Figure 12, the evolution of the ratios with the immersion time is similar between samples, with an increase on the first three days and a decrease on the seventh. The atomic ratio Ca/P was estimated by using four different areas in each sample, and the average and standard deviation (uncertainty) were calculated. The uncertainty values were similar for all samples and around 0.5. Although the value of the third day is unusually high (Ca/P » 3), the same trend in behavior is still perceived for all the samples. This trend is a clue that, at first, there is a calcium ion deposition taking place. After a maximum is reached (day 3), there is a significant decrease in the ratio.

Since the hydroxyapatite produced was calcium deficient, due to a Ca/P ratio of 1.5 before SBF immersion, it is possible that an initial deposition of Ca^2+^ ions is occurring, increasing the Ca/P ratio. In SEM images, no changes were observed at the surface of the samples (no flower-like apatitic crystals, aggregates of such crystals, or any continuous layer of CaP were detected). So, it is reasonable to assume that when the deposition of Ca^2+^ ions stops, due to lower concentration of ions in SBF and/or positive surface charges, the Ca^2+^ returns to the solution and PO_4_^3−^ ions become trapped at the surface. This results in the deposition of calcium ions, followed by their dissolution, and the deposition of phosphate ions, with no formation on the surface of apatitic structures. Also, at the maximum SEM magnification used, the crystals formed could be too small and not distinguishable from the original NW surface to be visible.

The recent literature reviews by Khare et al. [39] and Sood et al. [40] highlight studies involving hydroxyapatite-based materials incorporating particles of BT. Some research focuses on foams with similar porous sizes to the aerogels produced in this work and that showed enhanced bioactivity [41]. If the ceramics are electrically polarized, it is expected to improve further this important property [17,42]. In consequence, a better assessment of the advantages of using a HAp aerogel with BaTiO_3_ particles must include electrically polarized samples. Subsequently, future work on the aerogel will be made to test the bioactivity and, furthermore, in vitro assays will be performed to quantify cell adhesion and proliferation and the results of the polarized and the non-polarized material will be compared. On the other hand, as Park et al. observed [43], in vivo assays might immediately reveal the advantage of having a dipolar material for the non-polarized piezoelectric material developed.

## 4. Conclusions

The main goal of this work was to produce hydroxyapatite aerogels with and without BaTiO_3_ and to study their features (including bioactivity and biocompatibility) with the purpose of creating a new material with osteointegration potential.

The barium titanate powders that were used were tetragonal, and consequently piezoelectric and presenting permanent dipoles. A structural analysis verified that the samples synthesized at 180 °C for 18 h resulted in wirelike structures of crystalline AB-type carbonated hydroxyapatite. Additionally, an EDS analysis showed a calcium deficiency in HAp. The aerogel structures showed an average porosity of 99.68%, with interlinked HAp wires, and a relatively good distribution of BT particles. 

Cytotoxicity assays revealed that all the BT particles were non-cytotoxic for a concentration of 60 mg/mL. For the aerogel, a concentration range of 0.5–0.125 mg/mL for all samples showed cell proliferation above 100% and thus, they were biocompatible.

In the SEM images of bioactivity, it was difficult to differentiate between the possibly deposited apatite and the surface of the HAp wires. However, an EDS analysis showed an occurring trend of calcium ions deposition followed by its dissolution and the deposition of phosphate ions.

The HAp-based nanowire aerogels with piezoelectric nano- and microparticles of barium titanate were successfully produced and showed properties, such as bioactivity and biocompatibility, that point to a new biomaterial with osteointegration potential. The aerogel could be suitable for non-load-bearing applications, such as cavity filling. To fully disclose the bone regeneration potential of the composite, further studies are essential and must include electrical polarization, mechanical compressive behavior, and in vivo assays.

## Figures and Tables

**Table 1:** Crystallite size of each BaTiO_3_ sample according to the Scherrer equation.

Sample	Peak, 2*θ* (°)	FWHM, *β* (rad)	Crystallite Size, *τ* (nm)
280 nm	31.43	4.81 × 10^−3^	29.9
2 µm	31.54	4.42 × 10^−3^	32.5
3 µm	31.49	3.90 × 10^−3^	36.9

## Data Availability

Data are contained within the article and Supplementary Materials.

## Supplementary Materials

The following supporting information can be downloaded at: https://www.mdpi.com/article/10.3390/biomimetics9030143/s1, Protocol for SBF and Figure S1. DSC/TG for sample BaTiO_3_-280nm from 25 to 220 °C. Figure S2. SEM images of Hydroxyapatite synthesized at 120 °C for (a) and (b) 24 hours; (c) and (d) 30 hours. Figure S3. Quantifying EDS analysis of the HAp/BT280 aerogel after 7 days in SBF. Figure S4. Mapping EDS analysis of the HAp/BT280 aerogel after 7 days in SBF.
