# Supplementary material for: Bioactive Hydroxyapatite Aerogels with Piezoelectric Particles
Source: Biomimetics (Basel). 2024 Feb 27;9(3):143. doi: 10.3390/biomimetics9030143 (PMC10968090; doi:10.3390/biomimetics9030143)
Supplement: Supplementary file 1 [file biomimetics-09-00143-s001.zip › biomimetics-2759099-supplementary.pdf]

biomimetics

Article

Bioactive Hydroxyapatite Aerogels with Piezoelectric Particles

Catarina Tavares [1], Tânia Vieira [2], Jorge C. Silva [2,*], João P. M. R. Borges [1,*] and M. Carmo Lança [1]

[1] CENIMAT|i3N, Department of Materials Science, School of Science and Technology, NOVA University Lisbon, 2829-516 Caparica, Portugal; cdc.tavares@campus.fct.unl.pt (C.T.); mcl@fct.unl.pt (M.C.L.)
[2] CENIMAT|i3N, Department of Physics, School of Science and Technology, NOVA University Lisbon, 2829-516 Caparica, Portugal; ts.vieira@fct.unl.pt
* Correspondence: jcs@fct.unl.pt (J.C.S.); jpb@fct.unl.pt (J.P.M.R.B.)

Abstract: Open-cell foams based on hydroxyapatite (HAp) can mimic the extracellular matrix (ECM) to better replace the damaged hard tissues and assist in their regeneration processes. Aeorgels of HAp nanowires (NW) with barium titanate (BT) particles were produced for applications in bone regeneration. Considering the role of piezoelectricity in bone remodeling, all BT particles, of size 280 nm, 2 µm and 3 µm, contained $BaTiO_3$ in its piezoelectric tetragonal phase. The nanowires synthesized were verified to be AB-type carbonated hydroxyapatite. The aerogels showed high porosity and relatively homogeneous distribution of the BT particles. Barium titanate proved to be non-cytotoxic while all the aerogels produced were cytotoxic for an extract concentration of 1 mg/mL but became non-cytotoxic at concentrations of 0.5 mg/mL and below. It is possible that these results were affected by the higher surface area and quicker dissolution rate of the aerogels. In the bioactivity assays, SEM/EDS, it was not easy to differentiate between the apatite deposition and the surface of the HAp wires. However, a quantitative EDS analysis shows a possible CaP deposition/dissolution cycle taking place.

Keywords: Piezoelectricity; Bone Tissue Regeneration; Aerogel; Hydroxyapatite; $BaTiO_3$; Barium Titanate; Solvothermal Synthesis.

Citation: Tavares, C.; Vieira, T.; Silva, J.C.; Borges, J.P.M.R.; Lança, M.C. Bioactive Hydroxyapatite Aerogels with Piezoelectric Particles. *Biomimetics* **2024**, *9*, 143. https://doi.org/10.3390/biomimetics9030143

Academic Editor: Jing Sun

Received: 21 November 2023
Revised: 16 February 2024
Accepted: 19 February 2024
Published: 27 February 2024

Copyright: © 2024 by the authors. Licensee MDPI, Basel, Switzerland. This article is an open access article distributed under the terms and conditions of the Creative Commons Attribution (CC BY) license (https://creativecommons.org/licenses/by/4.0/).

Supplementary materials

Protocol for Simulated Body Fluid solution

For 1 liter of SBF, add the following reagents in the presented order to 750 mL of millipore water while stirring at 37 °C: 7.996 g of NaCl

0.35 g of $NaHCO_3$

0.224 g of KCl

0.228 g of $Na_2HPO_4.H_2O$

0.228 g of $MgCl_2.6H_2O$

15 mL of 1M HCl

0.0278 g of $CaCl_2.2H_2O$

0.0071 g of Na_2SO_4

6.057 g of $(CH_2OH)_3CNH_2$.

Measure the pH of the solution and, keeping the electrode submerged, add 1 M HCl drop by drop until the solution reaches a pH value of 7.4. Add millipore water to the solution until it reaches 1 liter. Keep the solution in a refrigerator for up to 1 month, heating it to 37 °C before using."

Figures

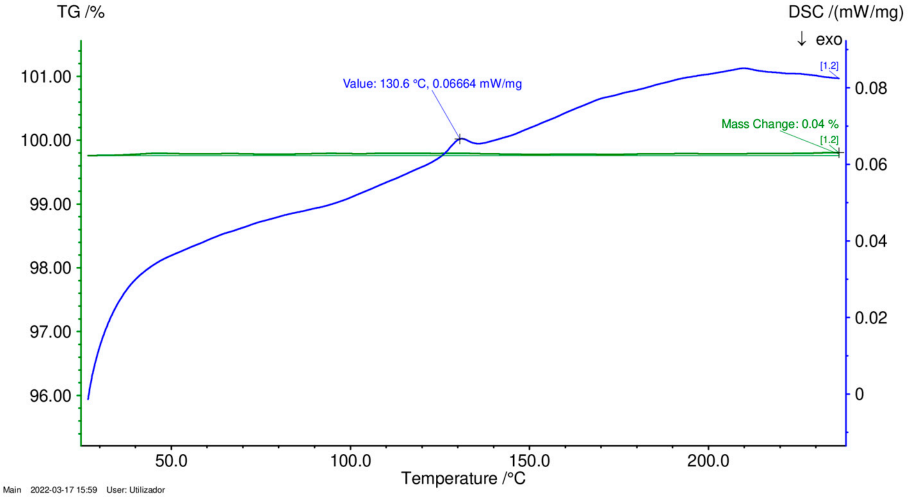

Figure S1. DSC/TG for sample BaTiO3-280nm from 25 to 220 °C.

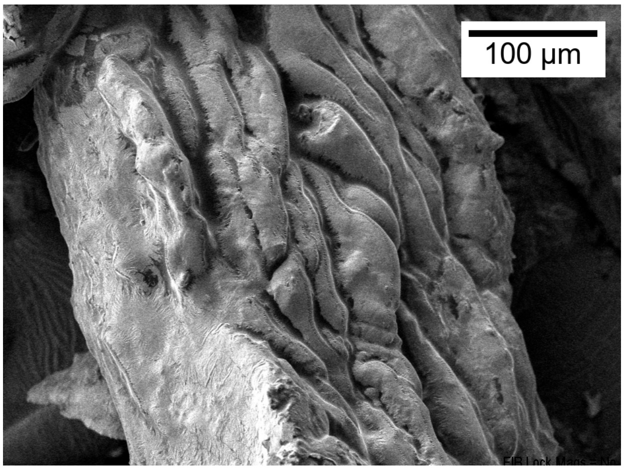

(**a**)

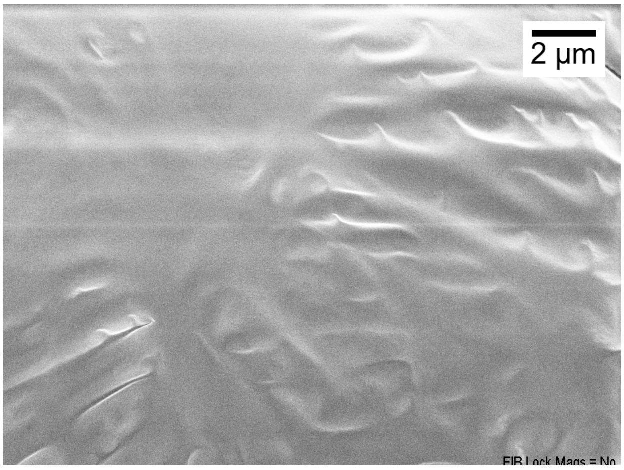

(**b**)

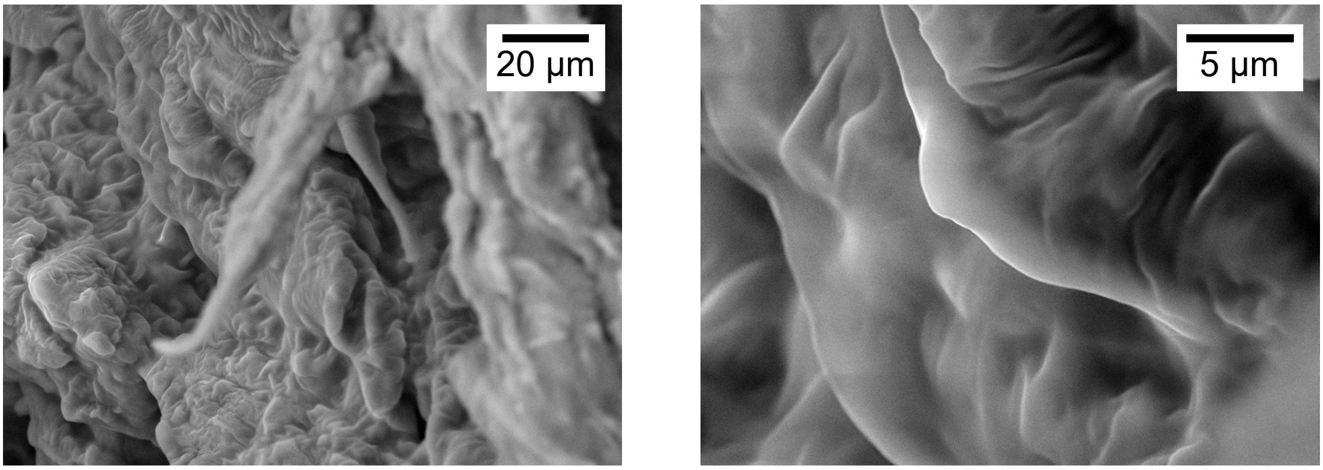

(**c**) (**d**)

Figure S2. SEM images of Hydroxyapatite synthesized at 120 °C for (a) and (b) 24 hours; (c) and (d) 30 hours.

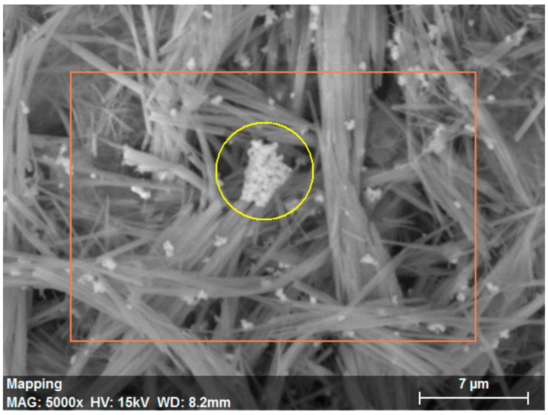

Element	C Atom. C [at.%]
Oxygen	49.89
Carbon	18.17
Calcium	12.30
Phosphorus	7.12
Titanium	3.71
Chlorine	3.50
Sodium	3.28
Barium	2.04

Figure S3. Quantifying EDS analysis of the HAp/BT280 aerogel after 7 days in SBF.

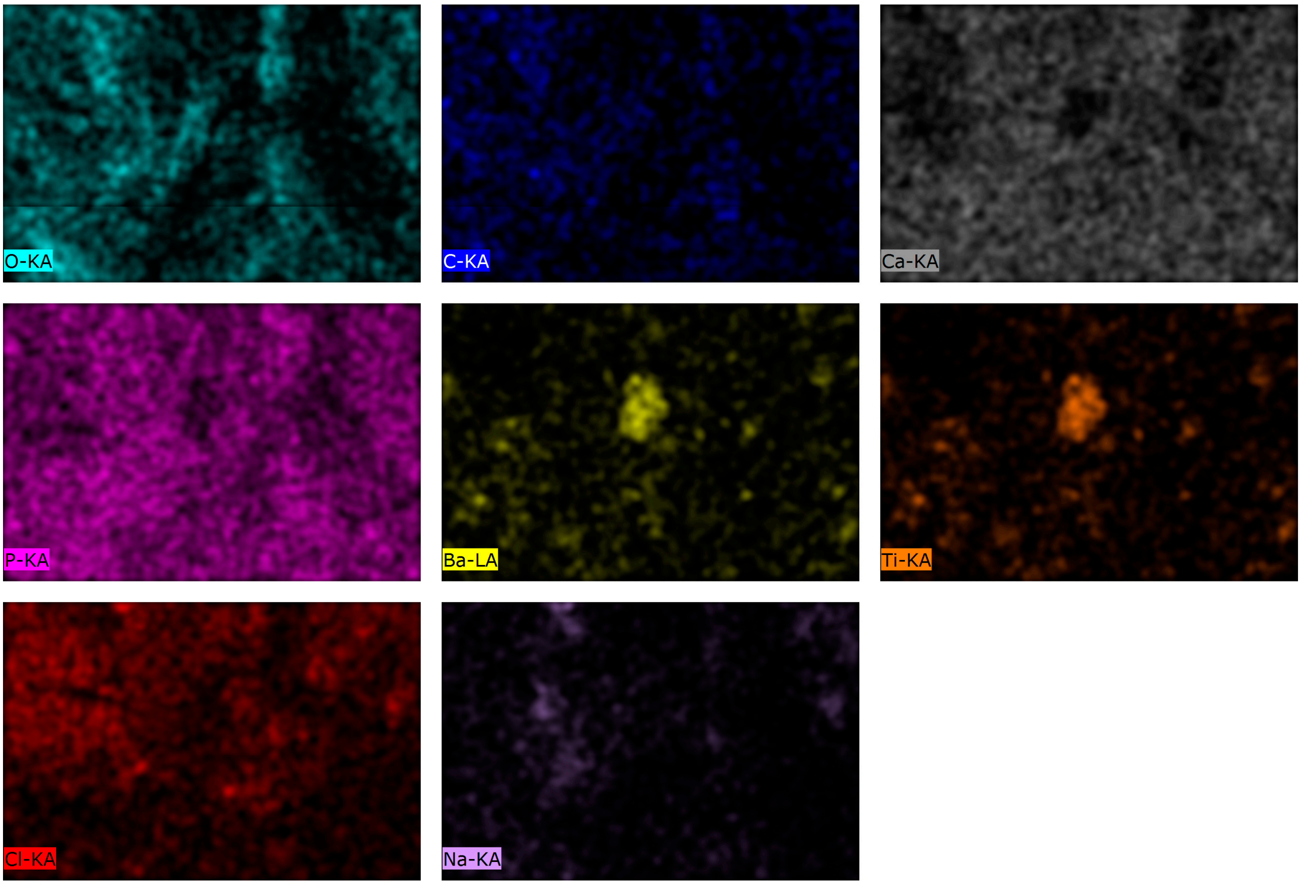

Figure S4. Mapping EDS analysis of the HAp/BT280 aerogel after 7 days in SBF.
